# Guide to Decomposition of Causal Effects Into Mediation, Interaction, and Direct Effects: Case Study on Aerobic Exercise and Parkinson Disease

**DOI:** 10.1212/WNL.0000000000209547

**Published:** 2024-06-10

**Authors:** Nina A. Hilkens, Gemma Hammerton, Nienke M. De Vries, Bastiaan R. Bloem, Yoav Ben-Shlomo, Sirwan K.L. Darweesh

**Affiliations:** From the Department of Neurology (N.A.H., N.M.D.V., B.R.B., S.K.L.D.), Radboud University Medical Centre; Donders Institute for Brain, Cognition and Behaviour (N.A.H., N.M.D.V., B.R.B., S.K.L.D.), Nijmegen, the Netherlands; Population Health Sciences (G.H., Y.B.-S.), Bristol Medical School, University of Bristol; Medical Research Council Integrative Epidemiology Unit at the University of Bristol (G.H.), Population Health Sciences, Bristol Medical School, University of Bristol, United Kingdom; and Center of Expertise for Parkinson & Movement Disorders (N.M.D.V., B.R.B., S.K.L.D.), Nijmegen, the Netherlands.

## Abstract

Mediation analysis can be applied in medical research with the aim of understanding the pathways that operate between an exposure and its effects on an outcome. This method can help to improve our understanding of pathophysiologic mechanisms and may guide the choice of potential treatment strategies. Traditional mediation analysis decomposes the total effect of an intervention on the outcome into 2 effects: (1) an indirect effect, from exposure using a mediator to the outcome, and (2) a direct effect, directly from exposure to outcome. A limitation of this method is that it assumes no interaction between the exposure and the mediator, which can either lead to an over- or underestimation of clinically relevant effects. The “4-way decomposition” method has the advantage of overcoming this limitation. Specifically, the total effect of an exposure on the outcome is decomposed into 4 elements: (1) reference interaction (interaction only), (2) mediated interaction (mediation and interaction), (3) the pure indirect effect (mediation but not interaction), and (4) the direct effect (no mediation and no interaction). We provide a guide to select the most appropriate method to investigate and decompose any causal effect given the research question at hand. We explain the application of the 4-way decomposition and illustrate this with a real-world example of how aerobic exercise may influence motor function in persons with Parkinson disease.

## Introduction

### Clinical Context

In clinical research, there is broad interest in understanding what mechanisms underlie causal associations between an exposure and an outcome. Such insight can be leveraged to improve our understanding of pathophysiologic mechanisms, which may in turn guide the choice of potential treatment strategies. Mediation analysis can be applied to unravel causal mechanisms, but it can be challenging for clinical researchers without specific expertise in this approach to identify an appropriate method to address their question at hand.

In this study, we provide a guide to help researchers select the most appropriate method to investigate and decompose any causal effect given the research question at hand. We first discuss the key causal concepts of mediation, effect modification, and interaction (Table 1) using examples from clinical research in the field of neurology. We then discuss a causal mediation framework that integrates these causal concepts and provide a real-world application of the framework to unravel how aerobic exercise may influence motor function in Parkinson disease (PD). Specifically, in our case study, we quantify how much of the total effect of aerobic exercise on motor functioning in PD is explained by an improvement in cardiorespiratory fitness through mediation, interaction, or both. The rationale and source data for this case study are outlined in Table 2.

**Table 1:** Definition of Terms

Term	Definition
Mediation	The effect of an exposure of interest on the outcome runs (in part) via a third variable, the mediator
Effect modification	The effect of an exposure of interest on the outcome differs by a third variable (the effect modifier), but the third variable itself has no effect on the outcome
Interaction	An exposure of interest and another variable (the interactor) both have an effect on the outcome, and the combination of the exposure of interest and the other variable has an additional (positive or negative) effect on the outcome

**Table 2:** Rationale and Source Data for a Case Study Examining Aerobic Exercise and PD Progression

**Rationale**
Converging evidence suggests that aerobic exercise improves motor functioning in people with PD.^21^ However, insight is scarce on causal mechanisms accounting for this improvement. Recent trials suggest that the total effect of aerobic exercise may be mediated, in part, by an improvement in cardiorespiratory fitness^22-24^ and, in part, by other mediators, such as a reduction in anxiety, depressive symptoms, and sleep impairments, which are assumed to not be directly determined by cardiorespiratory fitness.^25-27^
Intriguingly, the improvement in motor functioning is amplified in patients whose cardiorespiratory fitness increases most in response to aerobic exercise.^28^ This suggests that longitudinal improvement in cardiorespiratory fitness is not only a mediator of the effect of aerobic exercise but that there may also be interaction between aerobic exercise and cardiorespiratory fitness. In fact, there may be more than 1 interactive effect. On the one hand, an improvement in cardiorespiratory fitness may increase cerebral perfusion, which, in turn, may reduce motor impairments in PD, reflecting mediated interaction. On the other hand, aerobic exercise also promotes other mechanisms than cardiorespiratory fitness that may mediate the improvement, such as the induction of adaptive plasticity,^29^ reflecting reference interaction.
**Source data**
We performed a post hoc analysis on the Park-in-Shape study, which was a single-center, double-blind, home-based, randomized controlled trial comparing aerobic exercise with a nonaerobic intervention.^30^ The intervention in both groups was delivered in the patients' homes, with remote supervision by a coach (physical therapists or research assistant). Outcomes were assessed at baseline (baseline) and after 6 mo (follow-up), when the intervention was completed. The primary outcome was the between-group difference at follow-up in motor functioning, which was measured using the motor section score of the revised MDS-UPDRS part III^31^ in a standardized off state. Cardiorespiratory fitness was measured as the VO_2_max with graded maximal exercise testing. The trial protocol was approved by the medical ethical committee “Oost-Nederland.” The full protocol, including a detailed description of the intervention and of assessment methods, has been published,^30^ as have the results from the primary analysis.^16^

Abbreviations: MDS-UPDRS = Movement Disorders Society—Unified Parkinson's Disease Rating Scale; PD = Parkinson disease.

### Mediation

**Figure 1:**
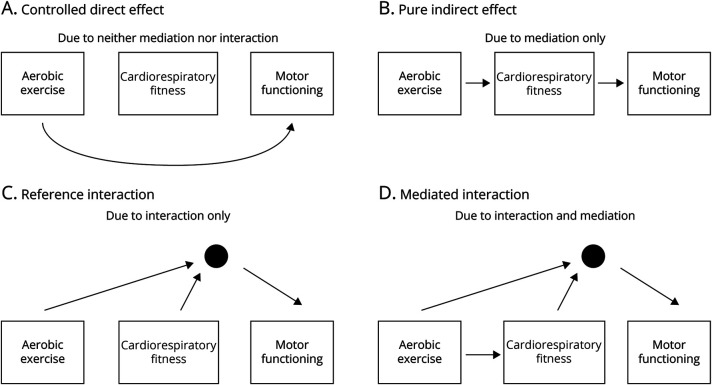
Decomposition of Total Effect of Exposure on Outcome The dot reflects an interaction term.

Mediation refers to the situation where (part of) the effect of an exposure (E) on an outcome (O) is explained by a mediator (M) (Table 1, Figure 1B). Researchers are usually interested, albeit oftentimes implicitly, in undertaking “mediation analysis” to investigate whether certain mechanisms (M) mediate the exposure-outcome (E-O) association and in quantifying the magnitude of these mechanisms. Understanding the biopsychosocial mechanisms underlying exposure-outcome associations, assuming causality, can help unravel pathophysiologic mechanisms and may allow improved iterations of the intervention that are more cost-effective and potentially better for affected individuals. Mediation analyses can also provide mechanistic insight into pharmacologic trials. For instance, if a drug alters pathways A and B and it can be shown that only pathway A mediates the benefits, then newer drugs specifically operating on pathway A may be more efficient, with potentially less side effects. Similarly, measuring the impact on mechanistic pathways can provide an intermediate biomarker that may be useful for monitoring therapy.

In our case study, the total effect of aerobic exercise may be mediated, in part, by an improvement in cardiorespiratory fitness and, in part, by other mediators, such as a reduction in anxiety, depressive symptoms, and sleep impairments, which are assumed to not be directly determined by cardiorespiratory fitness.

### Effect Modification

Having established that the exposure is associated with the outcome of interest, it is common to examine whether this association is the same across different population subgroups. If the effect differs across subgroups, there is evidence in support of “effect modification” (Table 1). Within the context of a randomized controlled trial (RCT), this is best undertaken by a prespecified subgroup analysis as part of the statistical analysis plan. In observational studies, this is often done implicitly when, for example, researchers present stratified results, for example, by sex or age group.

Results from subgroup analyses should be interpreted with caution because different subgroups have unequal abilities to detect effects because they contain different amounts of information, for instance, in terms of the distribution of relevant exposures, incidence of outcomes, or—in longitudinal studies—the person-time amassed. The presence or absence of effect modification also depends on the scale that is used to evaluate this. Therefore, claims of definitive differences in effects between subgroups should always be supported by a test for interaction. However, in some instances, sufficiently powered interaction analyses may not be feasible, for example, when only small sample sizes are available—as is often the case in studies on rare diseases. In such instances, it may be justified to highlight possible subgroup differences without an interaction test. The authors should then clarify that further research is warranted to establish definitive subgroup differences.

### Interaction

A related concept is to examine whether the effect of exposure (E) is influenced by another variable because of interaction (I) (Table 1, Figure 1C). Interaction generally refers to the situation where the joint effect of 2 variables is different from what would have been predicted by the combination of each of the individual effects. There are various types of interactions: (1) statistical interaction, (2) biological interaction, (3) interaction in public health, and (4) interaction in individual decision making. A further explanation of each type has previously been published elsewhere.^1,2^ Similar to the detection of effect modification, the detection of interaction also depends on which statistical model is used. Interaction can be established on an additive scale (e.g., when estimating mean differences or risk differences) or on a multiplicative scale (e.g., when estimating odds ratios or risk ratios).^3^

Interactions can be both positive and negative. In the first scenario, the combination of exposure (E) and interactor (I) leads to a greater risk of the outcome (O) than is expected from the sum of exposure (E) and interactor (I) alone (in the case of additive interaction). For example, let us assume that exposure to pesticides (E) has a modest effect in increasing the risk of PD, our outcome of interest. Let us also assume that individuals with genetic variants that are functionally associated with less efficient liver detoxification enzymes, and hence longer exposure to toxins (I), are also more likely to develop PD. Interaction would be observed whether individuals with both pesticide exposure and adverse genetic variants are disproportionally more likely to develop PD than would have been predicted based on the risks for each alone. In our case study, it is conceivable a priori that the beneficial effects of aerobic exercise and cardiorespiratory fitness on motor functioning in PD may interact. If that were to occur, then the improvement in motor functioning would be amplified in patients whose cardiorespiratory fitness increases most in response to aerobic exercise.

Far less common is the occurrence of negative interaction, where the combination of both exposure (E) and interactor (I) is associated with an observed risk that is lower than expected based on each exposure alone. An example of negative interaction can be found in the treatment of patients with chronic inflammatory demyelinating polyneuropathy. In these patients, intravenous immunoglobulin and plasmapheresis each has a beneficial effect on clinical outcomes when administered in isolation, but in combination, their effects wane because plasmapheresis removes the therapeutic immunoglobulin from the bloodstream.

In general, interactions are not only of interest because they may aid in unravelling mechanisms, but also because ignoring interactions in an analysis may result in misleading results and hence misinterpretation of the main effects.^4^ Conversely, a degree of caution is warranted when no evidence for interaction is observed as statistical power is generally low for these tests of interaction.

### Mediation and Interaction

A limitation of separately examining mediation and interaction is that it does not provide an accurate estimate in case a variable is both an interactor and a mediator. Traditional mediation approaches (e.g., product of coefficients)^5^ are limited by the fact that they assume no interaction between the exposure and the mediator and cannot accommodate nonlinear effects. To overcome these limitations, the counterfactual mediation framework has been developed. It allows noncontinuous mediators and outcomes as well as exposure-mediator interactions to be incorporated. In addition, the counterfactual framework has made the assumptions of mediation analysis much more explicit.^6^ To further disentangle the various pathways and quantify how much of the effect is due to mediation, interaction, or both, Vanderweele proposed the “4-way decomposition” (Figure 1). While we strongly encourage researchers interested in the 4-way decomposition to read the original publication,^7,8^ we discuss in the next paragraph the components of the framework and provide an introductory guide for clinical researchers who wish to understand the process of decomposing causal effects. The framework does not quantify effect modification because effect modifiers are not assumed to have a causal effect on the outcome.

### Four-Fold Decomposition: Conceptual Framework

Traditional mediation analysis simply dissects the total causal effect of an intervention on the outcome into 2 components. The first component is the direct effect of the exposure on the outcome, in the absence of the mediator or any interactor (*controlled direct effect*). The second component is the effect of the exposure on the outcome by using the mediator, without interaction (*pure indirect effect*). However, 2 further components can be added to our causal diagram. This becomes relevant if, for example, the benefits of the intervention reflect interactive effects of the exposure on the outcome. These effects can be either mediated by the mediator (*mediated interaction*) or purely because of interaction (*reference interaction*). The 4 components require different assumptions to be met regarding confounding. For the controlled direct effect, the model assumes that there are no unmeasured confounders for the E-O association and mediator-outcome associations. The other 3 components also require no unmeasured confounders for the exposure-mediator association and no mediator-outcome confounders that are affected by the exposure (eFigure 1).^7^

From these 4 effects, the overall percentage mediated is calculated as the sum of the pure indirect effect and mediated interaction divided by the total effect. The overall percentage attributable to interaction is calculated as the reference interaction plus mediated interaction divided by the total effect. The overall percentage eliminated is calculated as the total effect minus the controlled direct effect divided by the total effect. This percentage reflects how much of the effect of exposure on outcome could be eliminated by intervening on the mediator, which is relevant for policy making.^9^

Estimates of the 4 components can be obtained with linear regression analyses. The 4-way decomposition is flexible for different types of models including binary data, time-to-event data, and count data. Recently, software applications have been developed to implement this method in various software programs, including SAS, STATA, Mplus, and R.^10-12^

### Determining the Appropriate Analytical Plan

**Figure 2:**
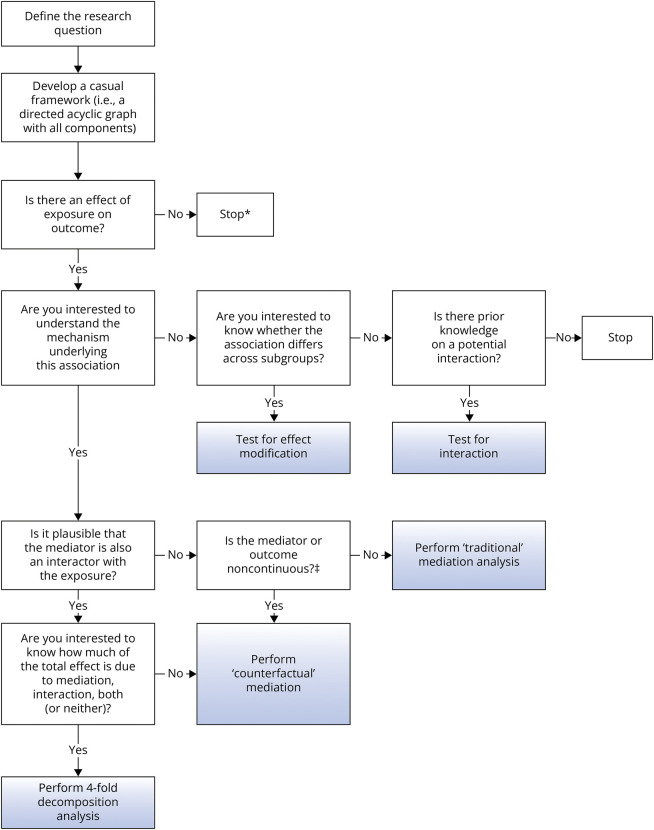
Flowchart to Guide the Choice of the Statistical Approach *An exception occurs if one is interested in effect modification irrespective of whether there is an overall effect of the exposure on the outcome. For example, if one is interested in the effects of an exposure on the outcome in certain subgroups. This should ideally be defined a priori in a study protocol. Rarely, researchers are interested in mediation even if there is no association between exposure and outcome, for example, when there is inconsistent mediation (direct and indirect effects may cancel out if they are in opposite directions). ‡Traditional mediation is valid with a rare binary outcome (10% is often used as a cutoff).

The question at hand determines which analytical technique is most appropriate. We have provided a flow diagram (Figure 2) to help logically consider the key issues and guide the researcher along the relevant analytical path. This flow diagram is intended to understand the mechanisms underlying associations that are a priori deemed to be causal, based on the association's strength, consistency, temporality, and other supportive criteria.^13^

Having established a possible causal association, there may be no interest in determining how this effect is mediated, whether the effects differ by subgroups, or whether the exposure interacts with other variables. Most RCTs are designed to simply detect a beneficial effect for all participants. Similarly, the evaluations of policy interventions, especially in public health, look at their impact at a global population level. If this is sufficient, then one would probably stop there analytically. For example, the introduction of banning smoking in public spaces has been shown to reduce risk of hospital admission for myocardial infarction, and from a policy perspective, this may be sufficient. However, *public health* is interested not only in effectiveness but also in whether interventions narrow or widen sociodemographic inequalities.^14,15^ Thus one may present the impact of the intervention depending on age group, sex, and socioeconomic status. These stratified results should be formally tested for effect modification. In addition, if you have a prior belief that the effectiveness of the policy interacts with a preexisting history of cardiovascular disease, then this additional analysis of interaction would be of interest. However, when you are interested in how the intervention works and you have measured a plausible mediating variable, then you will want to proceed to mediation analysis.

## An Exemplar Case Study: Statistical Analysis and Results

### Statistical Analysis

We hypothesized that if there is a total effect of aerobic exercise on motor functioning in PD, then this total effect can be decomposed into 4 effects (Figure 1). The first effect is mediated by change in cardiorespiratory fitness, without interaction between aerobic exercise and longitudinal change in cardiorespiratory fitness (*pure indirect effect*). The second and third effects reflect interactive effects of aerobic exercise and longitudinal change in cardiorespiratory fitness, which are either mediated by longitudinal change in cardiorespiratory fitness (*mediated interaction*) or purely interaction between aerobic exercise and cardiorespiratory fitness (*reference interaction*). The fourth effect is not mediated by—and does not interact with—longitudinal change in cardiorespiratory fitness (*controlled direct effect*).

We considered treatment group allocation as the exposure (E) of interest, VO_2_max at follow-up as the mediator (M), and Movement Disorders Society—Unified Parkinson's Disease Rating Scale (MDS-UPDRS) motor section score at follow-up as the outcome (O). We adjusted for the baseline values of VO_2_max and the MDS-UPDRS motor section score to test the differences after intervention. We assumed no confounding of the mediator-outcome pathway (which may or may not be true). The estimated total effect of aerobic exercise in the current report differs a little from the original publication because the main analysis of the Park-In-Shape trial^16^ was not adjusted for the baseline values of VO_2_max. The reason is that the aim of the main analysis was to quantify the total effect of aerobic exercise, whereas the aim of the current report was to decompose the causal effects of aerobic exercise.

We performed 2 separate sensitivity analyses to assess the robustness of the results, accounting for different scenarios of potential sources of confounding (eFigure 2). First, we repeated the analyses after additional adjustment for the remaining covariables from the main analysis in the original publication which we considered to be potential E-O confounders,^16^ that is, sex, treatment status, age, Hoehn and Yahr stage at baseline, and disease duration. Second, we repeated the analysis after removing the baseline values of VO_2_max from the main model.

Furthermore, we present estimates based on traditional mediation analysis (eFigure 3), in which the indirect effect is assessed based on the product of coefficients.^5^ The proportion that is mediated through VO_2_max is estimated by dividing the estimate of the indirect effect by the total effect. We have included the code of this exemplar case study as eMethods 1.

### Results

#### Baseline Values and Overall Effect

A total of 130 patients were included in the study, of whom 65 were randomized to aerobic exercise and 65 to a nonaerobic intervention. During 6 months follow-up, 20 patients did not adhere to their allocated exercise program (10 in each group). Furthermore, 5 patients did not complete the follow-up assessment. After exclusion of patients with missing values on MDS-UPDRS at baseline (n = 1), VO_2_max at baseline (n = 5), and VO_2_max at follow-up (n = 12), 114 remained for the analyses. At baseline, the mean VO_2_max score was similar in the intervention group (26.9 [SD 6.5]) and control group (26.3 [6.6]), whereas the MDS-UPDRS score was slightly higher in the intervention group (28.2 [13.2]) than in the control group (26.5 [14.8]). At follow-up, the mean VO_2_max score in the intervention group (28.4 [6.3]) was higher than in the control group (25.7 [7.1]) and the mean MDS-UPDRS score was lower in the intervention group (28.0 [11.7]) than in the control group (30.4 [15.0]). The adjusted between-group difference in the MDS-UPDRS score at follow-up was −3.8 (95% CI −6.3 to −1.2), which we consider the estimated overall effect of the exposure on the outcome. In the original study, this is reported as slightly larger at −4.2 (95% CI −6.9 to −1.6),^14^ reflecting the difference in analytical approach outlined in the previous section.

#### Traditional Analytical Methods

In the traditional mediation analysis (product of coefficients), the estimated indirect effect was 31% of the total effect. There was no strong evidence of an interaction between the allocated treatment and the follow-up value for VO_2_max (regression coefficient of interaction term −0.2, 95% CI −0.6 to +0.2) (eTable 1).

#### Four-Way Decomposition

The absolute effect of the exposure of −3.8 points on the outcome (indicating better motor function) was further decomposed, and the results indicated that most of this benefit was due to the controlled direct effect of the exposure of −2.7 points (95% CI −5.6 to +0.1) equivalent to 72% (95% CI 46%–146%) of the effect (Table 3). The mediated effect was more modest at 34% (95% CI −44% to 63%) and was mostly due to the pure indirect effect of −0.8 (95% CI −1.8 to +0.5) points (22%, 95% CI −42% to 48%) with a smaller mediated interaction effect of −0.4 (95% CI −1.3 to +0.4) (12%, 95% CI −23% to 35%). There was a very small reference interaction of +0.2 (95% CI −0.4 to +0.7) (−6%, 95% CI −20% to 18%; note that the proportion attributable to each component should generally not be calculated when effects are in opposing directions; we have done so here to provide an example).

**Table 3:** Four-Way Decomposition of the Effect of Aerobic Exercise on Motor Functioning, Treating Cardiorespiratory Fitness as Both a Mediator and a Interactor

Effect	Estimate^a^ (95% CI)	Percentage
Total effect	−3.8 (−6.3 to −1.2)	+100 (reference)
Controlled direct effect	−2.7 (−5.6 to +0.1)	+72 (46 to 146)
Reference interaction	+0.2 (−0.4 to +0.7)	−6 (−20 to 17)
Mediated interaction	−0.4 (−1.3 to +0.4)	+12 (−23 to 35)
Pure indirect effect	−0.8 (−1.8 to +0.5)	+22 (−42 to 48)

Abbreviations: MDS-UPDRS III = Movement Disorders Society—Unified Parkinson's Disease Rating Scale part III.

a Mean difference between aerobic exercise and anaerobic exercise groups in MDS-UPDRS III score at follow-up, adjusted for baseline MDS-UPDRS III score and baseline VO_2_max. Higher scores reflect greater severity of Parkinson disease motor impairments. Cardiorespiratory fitness is reflected by the VO_2_max, which measures maximal aerobic power. Higher scores reflect better cardiorespiratory fitness.

#### Sensitivity Analyses

The results were comparable after adjustment for additional confounders (eFigure 2). The percentage mediated by cardiorespiratory fitness decreased to 10% (95% CI −48% to 46%) in the model unadjusted for baseline values of VO_2_max, caused by a decrease in the percentage attributable to mediation only (pure indirect effect; 1% [95% CI −29% to 39%], −0.02 [95% CI −1.1 to 1.0] points) (eFigure 2).

## Discussion

In clinical neurologic research, we sometimes encounter situations in which the effect of an intervention is mediated by a variable that does not interact with the intervention. For instance, in the treatment of polyneuropathy, the effects of a neuropathic analgesic on quality of life are mediated by a reduction in pain, but it is unlikely that the effects of the analgesic and pain interact. However, in other situations, it is feasible that a mediator also interacts with the intervention. For instance, the effect of an antihypertensive drug (the intervention) to lower the risk of stroke (the outcome) may be mediated, in part, by stabilization of cerebral small-vessel disease, and the effects of the antihypertensive drug may also interact with the degree of cerebral small vessel disease. In this article, we present a guide to select the appropriate method to decompose a causal effect given the research question at hand. We also explain the application of the 4-way decomposition and illustrate this method with a real-world example from the field of PD.

We have provided a flow diagram to help logically consider the key issues and guide the researcher along the relevant analytical path. As an exemplar case study, we performed a post hoc 4-way decomposition analysis on data from a recent trial, which had previously shown a beneficial total effect of aerobic exercise on motor functioning in people with PD. In a traditional mediation analysis, we estimated that 31% of the effect of aerobic exercise was mediated by an improvement in cardiorespiratory fitness. In the 4-way decomposition framework, we estimated the mediated effect to be slightly higher (34%); however, the difference in these effect estimates should be interpreted with caution because of the wide confidence intervals. The results of the 4-way decomposition suggest that the effect of aerobic exercise is only weakly mediated by an improvement of cardiorespiratory fitness, implying that the effect is mediated by other factors such as a reduction in anxiety. We observed no evidence for interaction between aerobic exercise and an improvement of cardiorespiratory fitness.

We interpret these results with caution mainly because of the relatively small sample size of the Park-In-Shape trial, which was not originally designed for the current 4-way decomposition analysis. This precluded a precise quantification of causal effects, as illustrated by the wide confidence intervals of each effect. Because of the sample size, this study also lacked statistical power to quantify the effects of multiple mediators.^17^ Another limitation of our exemplar case study is that we could not verify that the effects of aerobic exercise on cardiorespiratory fitness preceded the effects of aerobic exercise and cardiorespiratory fitness on motor functioning, because we only had 2 measurements of aerobic exercise, cardiorespiratory fitness, and motor functioning. Therefore, we cannot rule out reverse causality (improved motor functioning led to improved fitness), although this seems unlikely. Despite these limitations, we observed intriguing hints for the potential of the 4-way decomposition framework to unravel causal effects.

The 4-way decomposition framework has the potential to disentangle causal effects of effective interventions, but has thus far only been applied in relatively few studies in clinical neurology.^18,19^ To date, the 4-way decomposition method has also only rarely been applied to data from RCTs.^20^ The assumption of control for mediator-outcome confounding is not needed to quantify the total effect of an intervention in a randomized trial, but it is needed for the decomposition of causal effects. The reason is that, in a trial, the exposure is randomized, but the mediator is not.^5^

Furthermore, our flow diagram may enable researchers without prior expertise on causal mediation analysis to identify an appropriate method to address their question at hand. Our exemplar case study highlights the potential of 4-way causal mediation analysis to decompose the effects of aerobic exercise in PD. In this example, the observation that the largest beneficial effects of exercise operate without improvement in aerobic fitness, putting aside issues around measurement of the mediator, highlights different pathways to be explored that either have different effects on dopaminergic transmission or indirectly through the impact of exercise on mood. Future studies with larger sample sizes can leverage this method to unravel causal effects of aerobic exercise and other therapies in PD.

### Data Sharing Statement

The dataset will be made available to qualified researchers upon reasonable request.

## Appendix 1: Authors

**Table:**

Name	Location	Contribution
Nina A. Hilkens, MD, PhD	Department of Neurology, Radboud University Medical Centre; Donders Institute for Brain, Cognition and Behaviour, Nijmegen, the Netherlands	Drafting/revision of the manuscript for content, including medical writing for content; study concept or design; analysis or interpretation of data
Gemma Hammerton, PhD	Population Health Sciences, Bristol Medical School, University of Bristol; Medical Research Council Integrative Epidemiology Unit at the University of Bristol, Population Health Sciences, Bristol Medical School, University of Bristol, United Kingdom	Drafting/revision of the manuscript for content, including medical writing for content
Nienke M. De Vries, PhD	Department of Neurology, Radboud University Medical Centre; Donders Institute for Brain, Cognition and Behaviour; Center of Expertise for Parkinson & Movement Disorders, Nijmegen, the Netherlands	Drafting/revision of the manuscript for content, including medical writing for content
Bastiaan R. Bloem, MD, PhD	Department of Neurology, Radboud University Medical Centre; Donders Institute for Brain, Cognition and Behaviour; Center of Expertise for Parkinson & Movement Disorders, Nijmegen, the Netherlands	Drafting/revision of the manuscript for content, including medical writing for content; major role in the acquisition of data
Yoav Ben-Shlomo, PhD, MRCP	Population Health Sciences, Bristol Medical School, University of Bristol, United Kingdom	Drafting/revision of the manuscript for content, including medical writing for content; study concept or design; analysis or interpretation of data
Sirwan K.L. Darweesh, MD, PhD	Department of Neurology, Radboud University Medical Centre; Donders Institute for Brain, Cognition and Behaviour; Center of Expertise for Parkinson & Movement Disorders, Nijmegen, the Netherlands	Drafting/revision of the manuscript for content, including medical writing for content; study concept or design; analysis or interpretation of data

## Glossary

MDS-UPDRS: Movement Disorders Society—Unified Parkinson's Disease Rating Scale
PD: Parkinson disease
RCT: randomized controlled trial
